# Panitumumab use in metastatic colorectal cancer and patterns of *RAS* testing: results from a Europe-wide physician survey and medical records review

**DOI:** 10.1186/s12885-017-3740-4

**Published:** 2017-11-28

**Authors:** J. Han van Krieken, George Kafatos, James Bennett, Laurent Mineur, Jiří Tomášek, Etienne Rouleau, Pavel Fabian, Giovanna De Maglio, Pilar García-Alfonso, Giuseppe Aprile, Parijan Parkar, Gerald Downey, Gaston Demonty, Jörg Trojan

**Affiliations:** 1 Radboud University Medical Center, Nijmegen, Netherlands; 2 Amgen Ltd, Uxbridge, UK; 3 Institute Sainte Catherine, Avignon, France; 4 Masaryk Memorial Cancer Institute, Faculty of Medicine, Masaryk University, Brno, Czech Republic; 5 Curie Institute, Paris, France; 6 University and General Hospital, Udine, Italy; 7 Gregorio Marañón Hospital, Madrid, Spain; 8 Amgen Ltd, Cambridge, UK; 9 Amgen GmbH, Zug, Switzerland; 10 University Hospital, Frankfurt, Germany

**Keywords:** Panitumumab, Metastatic colorectal cancer, mCRC, *RAS*, Physician survey, Medical records review

## Abstract

**Background:**

In Europe, treatment of metastatic colorectal cancer (mCRC) with panitumumab requires prior confirmation of *RAS* wild-type mutation status. Two studies – a physician survey and a medical records review (MRR) – were conducted to evaluate the use of panitumumab and awareness among prescribing oncologists of the associated *RAS* testing requirements in clinical practice.

**Methods:**

Both studies enrolled participants from nine European countries and were carried out in three consecutive rounds. Rounds 1 and 2 (2012–2013) examined *KRAS* (exon 2) testing only; the results have been published in full previously. Round 3 (2014–2015) examined full *RAS* testing (exons 2, 3, 4 of *KRAS* and *NRAS*) and was initiated following a change in prescribing guidelines, from requiring *KRAS* alone to requiring full *RAS* testing. For the physician survey, telephone interviews were conducted with oncologists who had prescribed panitumumab to patients with mCRC in the previous 6 months. For the MRR, oncologists were asked to provide anonymised clinical information, extracted from their patients’ records.

**Results:**

In Round 3, 152 oncologists and 131 patients’ records were included in the physician survey and MRR, respectively. In Round 3 of the physician survey, 95.4% (*n* = 145) of participants correctly identified that panitumumab should only be prescribed in *RAS* wild-type mCRC compared with 99.0% (*n* = 298) of 301 participants in Rounds 1 and 2, responding to the same question about *KRAS* testing. In Round 3 of the MRR, 100% (*n* = 131) of patients included in the study had confirmed *KRAS* or *RAS* wild-type status prior to initiation of panitumumab compared with 97.7% (*n* = 299) of 306 patients in Rounds 1 and 2 (*KRAS* only). Of those patients in Round 3, 83.2% (*n* = 109) had been tested for *RAS* status and 16.8% (*n* = 22) had been tested for *KRAS* status only.

**Conclusions:**

Physicians’ adherence to prescribing guidelines has remained high over time in Europe, despite the change in indication for panitumumab treatment, from *KRAS* to *RAS* wild-type mCRC. Additionally, this study demonstrates the uptake of full *RAS* testing among the majority of oncologists and pathologists.

**Electronic supplementary material:**

The online version of this article (10.1186/s12885-017-3740-4) contains supplementary material, which is available to authorized users.

## Background

The epidermal growth factor receptor (EGFR) is a cell surface protein that has become an important therapeutic target in colorectal cancer (CRC) [1]. Two monoclonal antibodies that target the extracellular domain of the EGFR have been developed: cetuximab (Erbitux), which is a recombinant immunoglobulin G1 mouse–human chimeric anti-EGFR monoclonal antibody (mAb), and panitumumab (Vectibix), a recombinant, fully human immunoglobulin G2 anti-EGFR mAb [1, 2]. Anti-EGFR therapy (treatment with cetuximab or panitumumab) has been shown to be effective in metastatic CRC (mCRC) [3–6]. Initially, patients with tumours that had mutations of exon 2 of the *KRAS* oncogene were found to be resistant to treatment with anti-EGFR mAbs [3, 7, 8]. Further studies provided evidence that additional mutations beyond *KRAS* exon 2 occurring in the wider *RAS* family of oncogenes, specifically in exons 3 and 4 of *KRAS* and exons 2, 3 and 4 of *NRAS*, are also predictive of a lack of response to anti-EGFR therapy [9–13].

Panitumumab was first approved in the European Union (EU) in December 2007 as monotherapy for the treatment of patients with mCRC and confirmed wild-type *KRAS* tumour status after failure of fluoropyrimidine-, oxaliplatin- or irinotecan-containing chemotherapy [3, 14]. However, in November 2011, the approved licence for panitumumab was extended to cover its use as a first-line agent in combination with 5-fluorouracil/folinic acid + oxaliplatin (FOLFOX) chemotherapy and as second line in combination with 5-fluorouracil/folinic acid + irinotecan (FOLFIRI) chemotherapy, again restricted to patients with confirmed wild-type *KRAS* tumour status [7, 15]. In June 2013, EU treatment guidelines changed to recommend that panitumumab should be prescribed to patients with mCRC and wild-type *RAS* tumour status (exons 2, 3, 4 of *KRAS* and *NRAS*), which should be confirmed prior to treatment initiation [16, 17]. The current label for panitumumab includes a contraindication for its use in combination with oxaliplatin-containing chemotherapy in patients with mutant or unknown *RAS* tumour status (or *KRA*S status before June 2013) [16].

Two studies – a physician survey and a medical records review (MRR) – were initiated in Europe in 2012 to evaluate physicians’ awareness of the correct therapeutic indication for panitumumab and to establish if it was being prescribed in accordance with this indication, which is to patients with mCRC and confirmed wild-type *KRAS* tumour status prior to treatment with panitumumab. The studies were carried out in three consecutive rounds; the results of the first two rounds of both studies have been published previously [18]. Overall in Rounds 1 and 2 of the physician survey, 298 (99.0%) of 301 physicians responded correctly that panitumumab should be administered only to patients with confirmed *KRAS* wild-type tumours. In Rounds 1 and 2 of the MRR study, 299 (97.7%) of 306 patients reportedly had confirmed wild-type *KRAS* status before the initiation of panitumumab treatment. Of 85 patients who were prescribed panitumumab with concurrent oxaliplatin-containing chemotherapy in Rounds 1 and 2 of the MRR, 83 (97.6%) had confirmed wild-type *KRAS* status before the initiation of treatment [18].

The results of the third round of the physician survey and MMR, which focused on full *RAS* testing*,* are presented here. The primary objective of Round 3 of the physician survey was to assess physicians’ knowledge of the updated indication for panitumumab treatment, following the changes to the label modifying its use from *KRAS* wild-type to *RAS* wild-type mCRC only. Similarly, for Round 3 of the MRR, the main aim was to estimate the prevalence of full *RAS* testing in the routine clinical management of patients being prescribed panitumumab.

## Methods

### Physician survey and MRR overview

The detailed methodology of Rounds 1 and 2 (assessing *KRAS* only) for both the physician survey and MRR, conducted from 2012 to 2013, has been published in full previously [18].

Rounds 3 of the physician survey and the MRR were carried out from September 2014 to November 2014 and September 2014 to June 2015, respectively. Physicians from the following nine European countries were invited to participate in the studies: Belgium, Czech Republic, Denmark, France, Germany, Italy, Spain, the Netherlands and Sweden.

For Round 3 of both studies, the following data sources were used to select a random sample of potential participants: (a) a medical marketing database provided by a healthcare industry provider (Cegedim S.A., Boulogne-Billancourt, France), filtered by specialty, and (b) a list of CRC physicians provided by local affiliates of the study sponsor (Amgen Ltd., Uxbridge, UK). Round 3 of the MRR was carried out by Amgen Ltd. and Round 3 of the physician survey was carried out by a separate industry provider (Adelphi Research, Bollington, UK).

### Eligibility criteria for the physician survey

Practising oncologists were included in Round 3 of the physician survey if they had treated at least three new or continuing patients with mCRC in the 3 months immediately preceding their invitation to participate in the survey, and only if they had prescribed panitumumab at least once during the previous 6 months. Potential participants were excluded if they had previously taken part in either Round 1 or Round 2 of the survey.

### Eligibility criteria for the MRR

Practising oncologists were included in Round 3 of the MRR if they had treated at least three new or continuing patients with mCRC in the 3 months immediately prior to receiving their invitation to participate. In addition, oncologists were only eligible if they had prescribed a first dose of panitumumab to treat a new patient with mCRC in the preceding 6 months. Again they were excluded if they had already taken part in either Round 1 or Round 2 of the MRR. In addition, only one oncologist per participating medical centre was permitted to participate in the MRR, in each round of the study.

Patients were eligible to be included in the MRR by their oncologist if they had received their first dose of panitumumab during the 6-month period before the time at which medical records were accessed for the study. As with participating oncologists, patients were excluded from the MRR if they had taken part in Rounds 1 or 2. Patients were also excluded if they were participating in an experimental clinical trial at the time of receiving panitumumab.

### Data collection

In Round 3 of the physician survey, telephone interviews were conducted with eligible oncologists using a standardised questionnaire (see Additional file 1 for interview guide) and following consistent data-collection procedures.

For each oncologist included in the MRR, the relevant anonymised information for eligible patients who had received their first dose of panitumumab was abstracted from their medical records using standardised forms. The oncologists were also asked to identify the pathology centre that performed the *RAS* (or *KRAS*) testing. Further information was then collected and reported by the pathologists at these centres, again using a standardised questionnaire.

### Statistical analysis

The data analysis was descriptive for both the physician survey and MRR. For the categorical study endpoints, the count and proportion (%) in each category, based on the appropriate denominator, were calculated. The 95% confidence intervals were calculated for the proportions based on a normal approximation to the binomial distribution.

## Results

### Physician survey

**Fig. 1:**
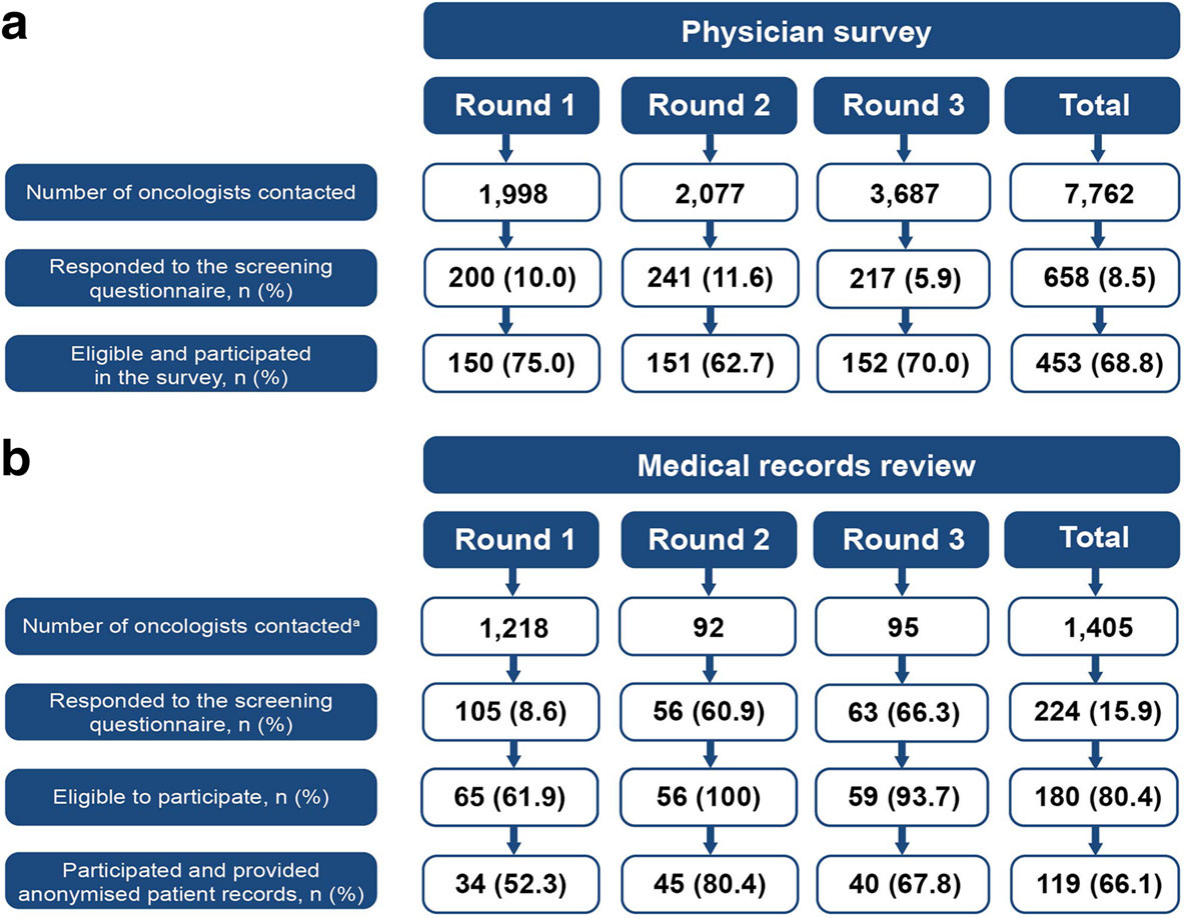
Physician disposition for the **a**) physician survey and **b**) medical records review studies. ^a^For Round 1, physicians were randomly selected for inclusion from a broad sampling list not filtered by speciality. A more targeted sampling of physicians filtered by speciality and based on the study eligibility criteria was used in Rounds 2 and 3

Across the nine participating European countries, a total of 3687 oncologists were contacted in Round 3 and sent an eligibility screening questionnaire. Of those approached, 217 oncologists responded to the screening questionnaire, resulting in a 5.9% response rate; of those responding, 152 (70.0%) were found to be eligible and subsequently participated in the survey (Fig. 1). The majority of participating oncologists were based in Germany, France, Italy and Spain. Comparatively few oncologists participated from the Czech Republic, Belgium, the Netherlands, Denmark and Sweden (Table 1).

**Table 1:** Oncologist characteristics in the physician survey and medical records review studies

	Physician survey			Medical records review		
Characteristic	Rounds 1 & 2 (*N* = 301)	Round 3 (*N* = 152)	Total (*N* = 453)	Rounds 1 & 2 (*N* = 79)	Round 3 (*N* = 40)	Total (*N* = 119)
Country, n (%)						
France	89 (29.6)	39 (25.7)	128 (28.3)	23 (29.1)	11 (27.5)	34 (28.6)
Germany	79 (26.2)	43 (28.3)	122 (26.9)	26 (32.9)	11 (27.5)	37 (31.1)
Italy	46 (15.3)	29 (19.1)	75 (16.6)	6 (7.6)	4 (10.0)	10 (8.4)
Spain	44 (14.6)	22 (14.5)	66 (14.6)	8 (10.1)	4 (10.0)	12 (10.1)
Czech Republic	18 (6.0)	6 (3.9)	24 (5.3)	5 (6.3)	3 (7.5)	8 (6.7)
Belgium	6 (2.0)	6 (3.9)	12 (2.6)	3 (3.8)	3 (7.5)	6 (5.0)
Denmark	5 (1.7)	2 (1.3)	7 (1.5)	3 (3.8)	1 (2.5)	4 (3.4)
Netherlands	9 (3.0)	3 (2.0)	12 (2.6)	2 (2.5)	1 (2.5)	3 (2.5)
Sweden	5 (1.7)	2 (1.3)	7 (1.5)	3 (3.8)	2 (5.0)	5 (4.2)
Type of institution, n (%)						
General or regional hospital	91 (30.2)	42 (27.6)	133 (29.4)	20 (25.3)	12 (30.0)	32 (26.9)
Oncology clinic/institute	30 (10.0)	9 (5.9)	39 (8.6)	7 (8.9)	5 (12.5)	12 (10.1)
Private clinic/hospital	50 (16.6)	25 (16.4)	75 (16.6)	33 (41.8)	11 (27.5)	44 (37.0)
University or teaching/training hospital	125 (41.5)	70 (46.1)	195 (43.0)	13 (16.5)	7 (17.5)	20 (16.8)
Other	5 (1.7)	6 (3.9)	11 (2.4)	6 (7.6)	5 (12.5)	11 (9.2)
Size of institution, no. of inpatient beds^a^	493.6 (465.6)	623.9 (434.5)	538.2 (475.6)	316.9 (525.4)	640.2 (570.7)	422.6 (559.0)
Mean (SD)	400.0	500.0	500.0	50.0	455.0	250.0
Median (Q1–Q3)	(100.0–772.5)	(310.0–895.5)	(150.0–800.0)	(17.0–400.0)	(250.0–750.0)	(23.0–625.0)
Size of oncology dept., no. of inpatient beds^a^					39.2 (51.4)	
Mean (SD)	NR	38.6 (58.5)	–	NR	30.0	–
Median (Q1–Q3)	NR	24.5 (14.0–45.0)	–	NR	(15.0–40.0)	–
No. of years’ experience as a practising oncologist specialising in mCRC						
Mean (SD)	12.5 (7.1)	13.1 (6.7)	12.7 (7.0)	15.7 (8.5)	13.9 (5.9)	15.1 (7.7)
Median (Q1–Q3)	11.0 (7.0–16.0)	12.0 (8.0–18.0)	12.0 (7.0–17.0)	15.0 (10.0–20.0)	14.0 (10.0–17.5)	15.0 (10.0–20.0)
No. of patients with mCRC treated by the oncologist in the previous 3 months						
Mean (SD)	59.0 (57.7)	53.1 (44.6)	57.1 (53.7)	58.0 (69.0)	37.5 (54.9)	51.1 (65.1)
Median (Q1–Q3)	40.0 (25.0–70.0)	40.0 (30.0–60.0)	40.0 (25.0–65.0)	36.0 (20.0–70.0)	28.0 (20.0–40.0)	30.0 (20.0–50.0)
No. of patients with mCRC treated with panitumumab in the last 6 months						
Mean (SD)	NR	NR	NR	9.2 (9.1)	6.7 (5.6)	8.4 (8.2)
Median (Q1–Q3)	NR	NR	NR	6.0 (5.0–10.0)	5.0 (3.0–9.0)	6.0 (5.0–10.0)

*mCRC* metastatic colorectal cancer, *NR* not recorded, *Q* quartile, *SD* standard deviation

^a^Rounds 1 and 2 of the study participants were asked only about the number of inpatient beds in their facility, which could have led to confusion regarding whether they should give the total number of beds in the hospital or in their oncology department. The survey was amended in Round 3 to prevent this confusion by specifically asking about both

In Round 3, the institutions with the highest number of participating oncologists were university and teaching/training hospitals (*n* = 70; 46.1%), followed by general or regional hospitals (*n* = 42; 27.6%) and private clinics and hospitals (*n* = 25; 16.4%). Among the study participants, the median duration of experience as a practising oncologist specialising in mCRC was 12.0 years (interquartile range [IQR] 8.0–18.0 years) and the median number of patients with mCRC who they had treated in the 3 months before their participation in the survey was 40.0 (IQR 30.0–60.0) (Table 1).

In Round 3, all 152 oncologists correctly identified that *RAS* testing should be performed prior to the initiation of panitumumab treatment. Furthermore, 145 (95.4%) gave the correct indication for panitumumab as being for the treatment of patients with mCRC and confirmed wild-type *RAS* tumour status (Table 2).

**Table 2:** Outcomes of *RAS* testing in Round 3 of the physician survey study

Outcome	No. of oncologists (%) (95% CI)
All oncologists	(*N* = 152)
Aware *RAS* testing should be performed prior to initiation of panitumumab	152 (100.0) (100.0–100.0)
Aware of the correct indication for panitumumab for treatment of patients with mCRC and wild-type *RAS* tumours^a^	145 (95.4) (92.1–98.7)
Aware of patients’ tumour *RAS* status prior to initiation of panitumumab treatment in the past 6 months of routine clinical practice^b^	143 (94.1) (90.3–97.8)
Administered panitumumab to only patients with mCRC and wild-type *RAS* in the past 6 months of routine clinical practice^c^	131 (86.2) (80.7–91.7)
Subset of oncologists who administered panitumumab concurrently with oxaliplatin-containing chemotherapy	(*N* = 105)
Administered panitumumab with concurrent oxaliplatin-containing chemotherapy to only patients with mCRC and wild-type *RAS* in the past 6 months of routine clinical practice^d^	97 (92.4) (87.3–97.5)

*CI* confidence interval, *mCRC* metastatic colorectal cancer

^a^Six oncologists responded for treatment of patients with mutant *RAS* tumours and one oncologist gave a ‘not sure’ response

^b^Eight oncologists were unaware of patients’ tumour *RAS* status before initiation of panitumumab treatment and one oncologist gave a ‘not sure’ response

^c^Nineteen oncologists had administered panitumumab to patients with mCRC and mutant *RAS* tumours or with unknown tumour *RAS* status, and two oncologists gave a ‘not sure’ response

^d^Eight oncologists had administered panitumumab with concurrent oxaliplatin-containing chemotherapy to patients with mCRC and mutant *RAS* tumours or with tumour *RAS* status unknown

When asked about tumour mutation testing, 48 (31.6%) oncologists indicated that all of their patients who were assessed for tumour mutation status in the preceding 6 months of routine clinical practice underwent full *RAS* testing. However, 46 (30.3%) oncologists indicated that their patients were tested for *KRAS* tumour mutation status only, and the remaining 58 (38.2%) indicated that while some patients were tested for *RAS* tumour mutation status, some patients had only been tested for *KRAS* tumour mutation status.

Prior to prescribing panitumumab in the past 6 months of routine clinical practice, 143 (94.1%) oncologists reported that they were aware of their patients’ tumour *RAS* mutation status. Only eight (5.3%) of the participating oncologists responded that they were not aware of their patients’ *RAS* tumour status before initiating panitumumab treatment, and one oncologist gave a ‘not sure’ response to the question (Table 2). Further to this, 19 (12.5%) oncologists responded that they had, in the past 6 months of routine clinical practice, administered panitumumab to patients, despite those patients having mutant or unknown *RAS* tumour status (Table 2). Of 13 (8.6%) oncologists who reported that they had administered panitumumab to patients with a known *RAS* tumour mutation, the most common reasons given for this action were ‘patient’s status or medical condition’ (*n* = 10; mainly observed as 'good patient condition) and ‘patient request’ (*n* = 3). Of seven (4.6%) oncologists who reported that they had administered panitumumab to patients with an unknown *RAS* tumour status, the most common reason given for this action was ‘time to obtain test results’ (*n* = 4) with reported times varying up to a month.

Of the 105 (69.1%) oncologists who had prescribed panitumumab simultaneously with oxaliplatin-containing chemotherapy, 97 (92.4%) confirmed that they had in the past 6 months of routine clinical practice only administered panitumumab simultaneously with oxaliplatin-containing chemotherapy to patients with confirmed wild-type *RAS* tumour status (Table 2).

In total, 118 (77.6%) of the oncologists surveyed in Round 3 recalled having received educational material regarding *RAS* testing, in the form of a physician education brochure detailing the importance of testing for *RAS* status.

### MRR

For Round 3 of the MRR, 95 oncologists were approached and sent the initial screening questionnaire. Of these, 63 (66.3%) responded to the screening questionnaire and 40 (42.1%) were eligible and agreed to participate in the study and provide anonymised information from their patients’ medical records (Fig. 1). Over half of the oncologists were from France and Germany; the rest were from Italy, Spain, the Czech Republic, Belgium, Sweden, Denmark and the Netherlands (Table 1).

The types of institutions with the highest number of participating oncologists were, in descending order, general or regional hospitals (*n* = 12; 30.0%), private clinics and hospitals (*n* = 11; 27.5%) and university or teaching/training hospitals (*n* = 7; 17.5%). The median duration of experience as a practising oncologist specialising in mCRC among participants was 14.0 years (IQR 10.0–17.5 years), and the median number of patients with mCRC who they had treated in the 3 months before their participation in the MRR was 28.0 (IQR 20.0–40.0) (Table 1).

The participating oncologists provided data for a total of 131 patients, who were then included in Round 3 of the MRR. The majority of these patients were male with a median age of 65.0 years (IQR 56.0–73.0 years). In addition to panitumumab treatment, 71 (54.2%) patients were also receiving concurrent oxaliplatin-containing chemotherapy (Table 3).

**Table 3:** Patient demographics in the medical records review study

	Rounds 1 & 2	Round 3	Total
All patients	(*N* = 306)	(*N* = 131)	(*N* = 437)
Sex – male, *n* (%)	204 (66.7)	85 (64.9)	289 (66.1)
Age (years) – mean (SD)	66.4 (10.9)	64.3 (11.3)	65.8 (11.1)
≥ 65 years, *n* (%)	189 (61.8)	69 (52.7)	258 (59.0)
≥ 75 years, *n* (%)	73 (23.9)	27 (20.6)	100 (22.9)
Patients receiving concurrent oxaliplatin^a^	(*N* = 85)	(*N* = 71)	(*N* = 156)
Sex – male, *n* (%)	65 (76.5)	49 (69.0)	114 (73.1)
Age (years) – mean (SD)	63.8 (11.2)	63.6 (10.1)	63.7 (10.6)
≥ 65 years, *n* (%)	48 (56.5)	33 (46.5)	81 (51.9)
≥ 75 years, *n* (%)	15 (17.6)	10 (14.1)	25 (16.0)

*SD* standard deviation

^a^Received oxaliplatin-containing chemotherapy during the interval from 7 days before the date of the first dose of panitumumab until 7 days after the last dose of panitumumab

Overall, 109 (83.2%) patients had been tested for *RAS* mutation status and 22 (16.8%) had only been tested for *KRAS* mutation status. However, before their first dose of panitumumab all 131 patients were tested for tumour mutation status and had tumours with either a confirmed wild-type *RAS* or *KRAS* mutation status. Of the 71 patients who were treated with concurrent oxaliplatin-containing chemotherapy, all had tumours with either a confirmed wild-type *RAS* or *KRAS* mutation status before their first dose of panitumumab (Table 4).

**Table 4:** Outcomes of *KRAS/RAS* testing in Round 3 of the medical records review study

Outcome	No. of patients (%)		
All patients	*RAS*(*N* = 109)	*KRAS*(*N* = 22)	Total(*N* = 131)
Tested for mutation status prior to first dose of panitumumab	109 (100.0)	22 (100.0)	131 (100.0)
Wild-type mutation status test result confirmed prior to first dose of panitumumab	109 (100.0)	22 (100.0)	131 (100.0)
Subset of patients treated with concurrent oxaliplatin-containing therapy	*RAS* (*N* = 64)	*KRAS* (*N* = 7)	Total (*N* = 71)
Tested for mutation status prior to first dose of panitumumab	64 (100.0)	7 (100.0)	71 (100.0)
Wild-type mutation status test result confirmed prior to first dose of panitumumab	64 (100.0)	7 (100.0)	71 (100.0)

Of the 28 pathology laboratories that were identified by participating oncologists in Round 3 of the MRR, 17 (60.7%) responded to the follow-up survey regarding their testing practices. All 17 laboratories had reportedly participated in at least one quality assurance (QA) scheme: seven (41.2%) had participated in the Directory of Molecular Genetics External Quality Assessment (EQA) Schemes; seven (41.2%) in a national or regional QA scheme (such as the Gen&Tiss scheme in France); six (35.3%) in the European Society of Pathology scheme; three (17.6%) in the United Kingdom National External Quality Assessment Service; two (11.8%) in the Quality Assurance Initiative of the German Society of Pathology; one (5.9%) in the College of American Pathologists; and one (5.9%) in another type of QA scheme. Of the 17 laboratories surveyed, 16 (94.1%) used a CE-marked or otherwise validated *RAS* mutation detection method (validation was performed in house, as per the International Organisation for Standardization 15,189 standard).

## Discussion

Recent changes to the prescribing guidelines for anti-EGFR mAbs (panitumumab and cetuximab) require *RAS* tumour genotyping to be carried out for patients with mCRC prior to the initiation of these therapies. These revisions have highlighted the need to gain a better understanding on prescribing oncologists’ awareness of these changes. The physician survey and MRR, the third rounds of which have been described here, were carried out to assess physicians’ knowledge of the updated indication for panitumumab treatment, following the changes to the label from *KRAS* to *RAS* mutation testing [18].

In Round 3 of the physician survey, all oncologists who participated were aware that *RAS* testing should be performed before their patients’ first dose of panitumumab. Further to this, nearly all of the oncologists (95.4%) also correctly identified that panitumumab is indicated for the treatment of mCRC in patients with confirmed wild-type *RAS* tumour status. These findings are consistent with the results of Rounds 1 and 2 of the physician survey, conducted in 2012–2013 before the latest label changes for panitumumab, where the majority (99.0%) of participants correctly identified that *KRAS* testing should be performed in patients with mCRC and confirmed wild-type *KRAS* tumours, in accordance with the then-correct indication for panitumumab [18].

In Round 3 of the MRR, all of the patients whose medical records were investigated had a confirmed wild-type tumour status before the initiation of panitumumab treatment, although a minority (16.8%) were only tested for *KRAS* mutation status. All patients included in the MRR who were treated with panitumumab and concurrent oxaliplatin-containing chemotherapy had confirmed wild-type tumour status, again though a small number (9.9%) were only tested for *KRAS* mutation status.

Nineteen of the oncologists (12.5%) who participated in Round 3 of the physician survey confirmed that they had administered panitumumab to at least one patient with mCRC and mutant or unknown *RAS* tumour status within the 6 months prior to completing the survey. The reasons given for these treatment decisions indicate that there may be clinical considerations relating to a patient’s clinical status, the practicalities of *RAS* testing, or the possibility that in later lines of therapy, patients and physicians may resort to treatments which are either not included in, or deviate from, guidelines. This suggests there is still a need for physician education which would enable each of these obstacles to be easily overcome and lead to improved practice so that all mCRC patients have a confirmed wild-type *RAS* tumour status before starting treatment with panitumumab.

In contrast to the physician survey, Round 3 of the MRR found that all patients studied had a confirmed wild-type tumour status prior to the initiation of panitumumab treatment; however, this was accounting for both *KRAS* and *RAS* testing, and the former was not explicitly asked about in the physician survey, which may in part explain the disparity. Furthermore, the physician survey and MRR results are not directly comparable, due to differences in the question regarding off-label prescription of panitumumab (the physician survey assessed the percentage of physicians who prescribed off-label to at least one patient in the last 6 months, and the MMR assessed the percentage of patients who were prescribed off-label panitumumab). These results were, again, broadly consistent with the combined results from Rounds 1 and 2 of both studies, which found that 5.0% of oncologists surveyed had treated a patient with panitumumab when they had either an unknown or mutant *KRAS* status while the MRR found that only 2.3% of patients had received panitumumab without having a confirmed wild-type *KRAS* status [18].

As other studies have shown, a minority of laboratories in Europe have continued to use *KRAS* testing since June 2013, despite the panitumumab label change [19]. This is important to note, both because of the update to the indication for anti-EGFR therapies and also because *KRAS* mutation testing is less sensitive than full *RAS* testing [20]. However, a further examination of the data from Round 3 of the MRR identified that 18 of the 22 samples tested for *KRAS* only had a test report date before the start of 2014, suggesting that they may have been carried out either before or immediately after the change to the prescribing guidelines. Additionally, in Round 3, each tumour sample was classified as having been tested for either *RAS* or *KRAS* based exclusively on the information recorded in the oncologist notes; the classification was not based on the specific exons and codons tested by the pathologist as this is often not recorded. As this is information which could not have been validated using another data source, it is possible there was some degree of misclassification with samples classified as *RAS* tested but in practice not tested for all exons 2, 3, 4 of *KRAS* and *NRAS*.

Despite efforts to obtain a higher response rate following the first two rounds of the study, the response rate for Round 3 was low (5.9%). This could potentially introduce selection bias as shown by the relatively high volume of mCRC patients treated by the participating oncologists (median of 40 in the past 3 months) [21, 22]. For the MRR study, a similarly low response rate was observed amongst oncologists in Round 1. A higher response rate was observed in Rounds 2 and 3 after changing to a more targeted methodology without this impacting the study results [18]. Finally, response rates of <10% are not uncommon for knowledge physician surveys [23].

As has been described elsewhere, *RAS* testing methods have been refined considerably over the last five years [24–26], and the results of Round 3 of the MRR are in agreement with this, showing that nearly all (94.1%) of the pathology laboratories surveyed regarding their *RAS* testing practices reported using a CE-marked or otherwise validated *RAS* mutation detection method and that all had participated in at least one QA scheme. However, there is still clear need for improvement, potentially via additional education, to ensure that all oncologists and pathologists treating patients with mCRC are implementing full *RAS* testing.

## Conclusions

The results presented here from Round 3 of the physician survey demonstrate that there is a high level of knowledge and awareness among practising oncologists regarding the need for full *RAS* testing in patients being considered for panitumumab treatment. The generally high awareness observed in the physician survey is also confirmed to an extent by Round 3 of the MRR, which provided insight into how this knowledge is being applied in routine clinical practice, and showed that the majority of patients are being tested for *RAS* tumour status before treatment initiation, but highlighted the fact that some patients are still only being tested for *KRAS* mutation status. It is important to underline that the use of panitumumab in patients with mutant or unknown *RAS* tumour status may be detrimental to patient outcomes and therefore, it is essential that oncologists follow the correct indication. Despite the change in guidelines after the introduction of the more comprehensive *RAS* testing, the Round 3 results showed that oncologists’ awareness and adherence to guidelines have remained high over time despite the change in guidelines and the introduction of the more comprehensive *RAS* testing.

## Additional files

Additional file 1: Interview guide/questionnaire used for the telephone interviews. (DOCX 61 kb)

Additional file 2: List of all Ethical committees that approved the study. (DOCX 16 kb)

## Abbreviations

CRC: Colorectal cancer
EGFR: Epidermal growth factor receptor
EQA: External quality assessment
EU: European union
FOLFIRI: 5-fluorouracil/folinic acid + irinotecan
FOLFOX: 5-fluorouracil/folinic acid + oxaliplatin
IQR: Interquartile range
mAb: Monoclonal antibody
mCRC: Metastatic colorectal cancer
MRR: Medical records review
QA: Quality assurance
