# Supplementary material for: Panitumumab use in metastatic colorectal cancer and patterns of RAS testing: results from a Europe-wide physician survey and medical records review
Source: BMC Cancer. 2017 Nov 28;17:798. doi: 10.1186/s12885-017-3740-4 (PMC5706421; doi:10.1186/s12885-017-3740-4)
Supplement: Supplementary file 1 — Interview guide/questionnaire used for the telephone interviews. (DOCX 61 kb) [file 12885_2017_3740_MOESM1_ESM.docx]

| ***Sponsor***  **A**  One Amgen Center Drive  Thousand Oaks, CA 91320 – 1799  (805) 447 - 1000 | Protocol Number  **Vectibix^®^**  **20101121** |
| --- | --- |

**CASE REPORT FORMS**

**PHYSICIAN SURVEY**

| **Survey of Oncologists in Europe to Evaluate Their Knowledge of *RAS* Testing** |
| --- |

***INSTRUCTIONS FOR COMPLETING***

***CASE REPORT FORMS***

|  |  | The CASE REPORT FORMS must be completed in **ENGLISH** | |
| --- | --- | --- | --- |
|  |  | Type or print using only **BLACK BALLPOINT INK** | |
|  |  | Corrections should be made **ONLY** as follows: | |
|  |  |  | Draw a single line through the incorrect entry |
|  |  |  | Enter correct data |
|  |  |  | Initial and date the correction |
|  |  |  | **DO NOT ERASE, WRITE OVER, OR USE CORRECTION FLUIDE OR CORRECTION TAPE** |
|  |  | Do not write in shaded areas. | |
|  |  | Add comments to Comment and *“Specify”* fields only | |

| **A** | **Vectibix^®^ 20101121** |
| --- | --- |

**Table of Contents**

| [Eligibility Worksheet 2](#_Toc312301766)  [Survey of Oncologists in Europe to Evaluate their Knowledge of *RAS* Testing 3](#_Toc312301767) |
| --- |

# Eligibility Worksheet

***Inclusion Criteria***

***Note: For any of the following questions: If the response is No the oncologist will not be able to participate in the 20101121 study.***

| **Criteria Number** | **Inclusion Criteria** | **Yes** | **No** | **Not Applicable** |
| --- | --- | --- | --- | --- |
| 101 | Is the physician a practicing oncology specialist? |  |  |  |
| 102 | ~~Has the oncologist treated at least 5 patients in the last 3 months with metastatic colorectal cancer?~~  (Note: only applies to Round 1) |  |  |  |
| 103 | Has the oncologist prescribed Vectibix (Panitumumab) within the last 6 months for metastatic colorectal cancer? |  |  |  |
| 104 | Has the oncologist indicated a willingness to participate in the Survey of Oncologists in Europe to Evaluate their Knowledge of *RAS* Testing, Amgen Study 20101121? |  |  |  |
| 105 | Has the oncologist treated at least three new or continuing patients in the last 3 months with metastatic colorectal cancer?  (Note: only applies to Rounds 2 and 3) |  |  |  |

***Exclusion Criteria***

***Note: For any of the following questions: If the response is Yes, the oncologist will not be able to participate in the 20101121 study.***

| **Criteria Number** | **Exclusion Criteria** | **Yes** | **No** | **Not Applicable** |
| --- | --- | --- | --- | --- |
| 201 | Has the oncologist participated in either the current round or any previous round of the 20101121 Survey Study? |  |  |  |
| 202 | ~~Has the oncologist participated in either the current round or any previous round of the 20101120 Medical Record Review Study?~~  (Note: only applies to Rounds 1 and 2) |  |  |  |

***Eligibility verified by_________________________ Date_______________***

# Survey of Oncologists in Europe to Evaluate their Knowledge of *RAS* Testing

| 1. | When should *RAS* testing be performed for metastatic colorectal cancer patients who receive Vectibix for the first time?  *(Please check only one answer)* | |
| --- | --- | --- |
|  |  | Prior to initiation of Vectibix |
|  |  | During treatment with Vectibix |
|  |  | After disease progression following treatment with Vectibix |
|  |  | *RAS* testing is not appropriate for these patients |
|  |  | Not Sure |

| 2. | Which of the following statements is correct regarding the current indication for Vectibix in patients with metastatic colorectal cancer? *(Please check only one answer)* | |
| --- | --- | --- |
|  |  | Treatment of patients with *mutant RAS tumors* |
|  |  | Treatment of patients with *wild type RAS tumors* |
|  |  | Treatment of patients where tumor *RAS* mutation status is unknown |
|  |  | Not sure of the current Vectibix indication with regard to tumor *RAS* mutation status |

| 3. | Did you prescribe Vectibix in the past 6 months in one or more of the following scenarios for the treatment of metastatic colorectal cancer?  (*Please check all that apply)* | |
| --- | --- | --- |
|  |  | In first line in combination with FOLFOX |
|  |  | In second line in combination with FOLFIRI for patients who have received first-line fluoropyrimidine-based chemotherapy? |
|  |  | As monotherapy after failure of fluoropyrimidine, oxaliplatin and irinotecan containing chemotherapy regimens? |

| 4. | In the past 6 months of routine clinical practice, have you always been aware of your patients’ tumor *RAS* mutation status prior to the initiation of Vectibix treatment?  *(Please check only one answer)* | |
| --- | --- | --- |
|  |  | Yes |
|  |  | No |
|  |  | Not Sure |

| 5. | In the past 6 months of routine clinical practice, what testing has been used to determine your patients’ tumor mutation status prior to the initiation of Vectibix treatment?  *(Please check only one answer)* | |
| --- | --- | --- |
|  |  | Patients tested for *RAS* tumor mutation status only |
|  |  | Patients tested for *KRAS* tumor mutation status only  Some patients tested for *RAS* tumor mutation status and some patients tested for *KRAS* mutation status |
|  |  | Unaware of the need for testing of patients’ tumor mutation status |
|  |  | Not sure |

| 6. | In the past 6 months of routine clinical practice, have you administered Vectibix to metastatic colorectal cancer patients with *RAS* mutant tumors?  *(Please check only one answer)* | |
| --- | --- | --- |
|  |  | Yes |
|  |  | No |
|  |  | Not Sure  *(If No or Not Sure please go to Question 8.)* |

| 7. | In the past 6 months of routine clinical practice, what factors have influenced your decisions to administer Vectibix to metastatic colorectal cancer patients with *RAS* mutant tumors?  (*Please check all that apply)* | |
| --- | --- | --- |
|  |  | Patient’s status or medical condition, please specify in as much detail as possible: ______________________­­­___________ |
|  |  | Patient request, please specify in as much detail as possible: _________________ |
|  |  | Not aware of the relevance of tumor *RAS* mutation status to Vectibix administration |
|  |  | Not Sure |
|  |  | Other, please specify in as much detail as possible:___________________________ |

| 8. | In the past 6 months of routine clinical practice, have you administered Vectibix to metastatic colorectal cancer patients with tumor *RAS* mutation status unknown?  *(Please check only one answer)* | |
| --- | --- | --- |
|  |  | Yes |
|  |  | No |
|  |  | Not Sure  *(If No or Not Sure please go to Question 10.)* |

| 9. | In the past 6 months of routine clinical practice, what factors have influenced your decisions to administer Vectibix to metastatic colorectal cancer patients with tumor *RAS* mutation status unknown?  (*Please check all that apply)* | |
| --- | --- | --- |
|  |  | Cost to perform testing |
|  |  | Time to obtain authorization to perform testing, please specify in as much detail as possible:_______________________________________ |
|  |  | Time to obtain test results, please specify in as much detail as possible:___________ |
|  |  | Tissue not available / Insufficient tissue available |
|  |  | Patient’s status or medical condition, please specify in as much detail as possible: ________________________ |
|  |  | Patient request, please specify in as much detail as possible___________________ |
|  |  | Unaware of the need for testing of patients’ mutation status |
|  |  | Not Sure |
|  |  | Other, please specify in as much detail as possible:___________________________  *(Note to the surveyor: Please ensure any responses given for Questions 8 and 9 are consistent with the response in Question 4.)* |

| 10. | In the past 6 months of routine clinical practice, have you administered Vectibix simultaneously with oxaliplatin-containing chemotherapy to metastatic colorectal cancer patients?  *(Please check only one answer)* | |
| --- | --- | --- |
|  |  | Yes |
|  |  | No |
|  |  | Not Sure |
|  |  | *(If No or Not Sure please go to Question15.)* |

| 11. | In the past 6 months of routine clinical practice, have you administered Vectibix simultaneously with oxaliplatin-containing chemotherapy to metastatic colorectal cancer patients with *RAS* mutant tumors?  *(Please check only one answer)* | |
| --- | --- | --- |
|  |  | Yes |
|  |  | No |
|  |  | Not Sure  *(If No or Not Sure please go to Question13.)* |

| 12. | In the past 6 months of routine clinical practice, what factors have influenced your decisions to administer Vectibix simultaneously with oxaliplatin-containing chemotherapy to metastatic colorectal cancer patients with *RAS* mutant tumors?  *(Free text response required)* ______________________________________ |
| --- | --- |

| 13. | In the past 6 months of routine clinical practice, have you administered Vectibix simultaneously with oxaliplatin-containing chemotherapy to metastatic colorectal cancer patients with tumor *RAS* mutation status unknown?  *(Please check only one answer)* | |
| --- | --- | --- |
|  |  | Yes |
|  |  | No |
|  |  | Not Sure  *(If No or Not Sure please go to Question15.)* |

| 14. | In the past 6 months of routine clinical practice, what factors have influenced your decisions to administer Vectibix simultaneously with oxaliplatin-containing chemotherapy to metastatic colorectal cancer patients with tumor *RAS* mutation status unknown?  *(Free text response required)* ______________________________________ |
| --- | --- |

| 15. | Have you ever received Vectibix education material regarding *RAS* mutation testing?  *(Please check only one answer)* | |
| --- | --- | --- |
|  |  | Yes |
|  |  | No |
|  |  | Not Sure  *(If Yes, please complete Question16.)*  *(If No or Not Sure, please STOP)* |

| 16. | When did you last receive this material? (yyyy) | │_│_│_│_│ |
| --- | --- | --- |

|  | | | |
| --- | --- | --- | --- |
| ***The following sections are to be completed by the surveyor*** | | | |
| During the telephone survey, did the oncologist volunteer details that a patient or patients may have had an adverse event (AE) or serious adverse event (SAE)? | | | |
|  |  | Yes | |
|  |  | No | |
|  |  | *If Yes, please complete the Amgen AE/SAE form and submit to Amgen Global Safety within 24 hours.* | |
| Date Survey Completed (dd-mmm-yyyy) | | | │_│_│/│_│_│_│/│_│_│_│_│ |

*Oncologists survey verified by ______________________ Date_____________*
