# Supplementary material for: Panitumumab use in metastatic colorectal cancer and patterns of RAS testing: results from a Europe-wide physician survey and medical records review
Source: BMC Cancer. 2017 Nov 28;17:798. doi: 10.1186/s12885-017-3740-4 (PMC5706421; doi:10.1186/s12885-017-3740-4)
Supplement: Supplementary file 2 — List of all Ethical committees that approved the study. (DOCX 16 kb) [file 12885_2017_3740_MOESM2_ESM.docx]

**Additional file 2**

# Ethics approval and consent to participate – ethical committees

# The study was approved by the following ethical committees (ECs): EC der Bayerischen Landesärztekammer (Germany); EC Azienda AOU S. Maria della Misericordia di Udine, EC dell’IRCCS Istituto Dermatologico dell’Immacolata – Ospedale Generale S. Carlo di Roma, EC AOU S. Giovanni Battista di Torino, EC dell’Università Campus Biomedico di Roma, EC Ospedali Riuniti di Ancona, EC dell’INT di Milano, EC dell’Ospedale San Filippo Neri di Roma, and EC per la sperimentazione Clinica della provincia di Treviso (Italy); EC de Investigacion Clinica H.G.U. Gregorio Maranon (Spain); EC Masarykova Onkologickeho Ustavu, Brno, etické komise pro multicentrické klinická hodnocení Krajská nemocnice Liberec, and etické komise pro multicentrické klinické hodnocení Motol (Czech republic), Medical EC des Cliniques Saint-Joseph, Liege (Belgium), and the Regional EC Board in Sweden—Karolina Institutet/ Solna.
